# A Case of Tooth Impacted in the Left Bronchus: Gangrene of the Left Lung: Death

**Published:** 1899-10

**Authors:** James S. Warrack

**Affiliations:** Demonstrator of Physiology in the University of Aberdeen, in British Medical Journal


ARTICEL XII

### A CASE OF A TOOTH IMPACTED IN THE LEFT BRONCHUS: GANGRENE OF THE LEFT LUNG: DEATH.

A women, aged 26, was admitted to the Victoria Hospital, Burnley, on December 18th, and died on December 30th, 1897. She stated, on admission, that four days previously she was given gas for the purpose of having a tooth extracted. At the critical moment she appears to have taken a deep breath, the result being that the tooth, which must have escaped from the forceps, was inhaled into the air passages. Thereafter extreme cyanosis occurred, and she had a feeling of tightness in the throat, with dyspnea, and a hard dry cough, aggravated by speaking or change of position. She had also a vague feeling of something being fixed inside the chest, but could not indicate the position. When I saw her she was lying on her back, with livid lips, and coughing in frequent paroxysms, but without expectoration. The coughing was much aggravated by attempts to speak, and by change of posture. The left side of the chest scarcely moved at all on respiration. There were no breath sounds audible over the left side of the chest, with the exception of tubular breathing at the left apex, accompanied by small moist *rules*, and this was indistinct posteriorly. On the outside of the left nipple there was a small area over which a friction rub was audible. On the left side, from the level of nipple down to an inch from the umbilicus and backwards as far as the mid-axillary line, there was a well-marked area of hyperesthesia, which corresponded roughly to the distribution of the sixth, seventh and eighth dorsal spinal roots. The breath sounds were harsh on the right side but otherwise normal. The pulse and respiration-rates were much quickened. During the next few days there was little alteration in the patient's condition, but the temperature assumed the hectic type. On December 27th, thirteen days after the accident, there were-marked signs of gangrene of the left lung. The breath was offensive, and there was expectoration of a brown and foul-smelling sputum. The lower cervical glands on the left side were enlarged, and there was a well-marked friction rub in the left axilla. Her condition became gradually worse until death occurred on December 30th. At the necropsy on December 31st, 1897, I explored the trachea up to the larynx, but no foreign body was found there. All over the left pleural cavity there were numerous recent adhesions, and on separating these some grumous fluid came away from the lung. The heart was flabby and rather smaller than usual, and its right side was dilated. On cutting open the left bronchas the missing tooth was found. It was tightly wedged in the bronchus, its point being downwards and the crown upwards, so that it had apparently acted as a "ball valve." It had almost ulcerated through the left bronchus, which above the situation of the tooth was congested and full of grumous fluid. On following the left bronchus into the lung there was found an irregular passage full of a brown smelling fluid. The whole of the left lung was gangrenous and creptitating, and on squeezing the lung substance there exuded a fluid of the character described. The bronchial glands were enlarged, and there was also an enlarged gland at the root of the neck on the left side. Above the impacted tooth no bronchiole was given off, so that there must have been entire obstruction. The right lung was congested and edematous, showed commencing consolidation at the base. There were two cavities in the left lower jaw, and into the hinder one the tooth, which was carious, fitted exactly, its direction being forwards and inwards. This case seems to illustrate the effects of obstruction to a bronchus, as Dr. Sevestre has recently pointed out in the *British Medical Journal*, the early stage being collapse of the lung, and the later, inflammation, leading sometimes to the entire disorganization and gangrene of the lung. The tooth did not appear to have been tightly wedged in at first when the patient was seen, but seemed to have become so owing to the incessant attempts to expel the foreign body, and with each attempt the conditions must have been aggravated by the suction in during inspiration. The gangrene would be excited both by the retention of secretion, and by the introduction of septic material from the tooth.—*James S. Warrack, M. A., M. D., Demonstrator of Physiology in the University of Aberdeen, in British Medical Journal, Feb. 18th, 1899.*

